# Preparation and characterization of small-diameter decellularized scaffolds for vascular tissue engineering in an animal model

**DOI:** 10.1186/s12938-017-0344-9

**Published:** 2017-05-11

**Authors:** Shuangyue Xu, Fangna Lu, Lianna Cheng, Chenglin Li, Xu Zhou, Yuan Wu, Hongxing Chen, Kaichuang Zhang, Lumin Wang, Junjie Xia, Guoliang Yan, Zhongquan Qi

**Affiliations:** 1 Organ Transplantation Institute of Xiamen University, Xiamen, 361102 Fujian Province People’s Republic of China; 2 Fujian Key Laboratory of Organ and Tissue Regeneration, Xiamen, 361102 Fujian Province People’s Republic of China; 3 Medical College, Xiamen University, Xiamen, 361000 Fujian Province People’s Republic of China; 4 Cardiovascular Surgery, Heart CenterXiamen University Affiliated Zhongshan Hospital, Xiamen City, 361000 Fujian Province People’s Republic of China; 5 Basic Medical Department of Medical College, Xiamen University, Xiamen, 361102 Fujian Province People’s Republic of China; 6 Departmant of Neurosurgery, Fuzhou Second Affiliated Hospital of Xiamen University, Fuzhou, 350007 Fujian Province People’s Republic of China; 7 Department of Laboratory Medicine, Lishui People’s Hospital, Lishui, 323000 Zhejiang People’s Republic of China

**Keywords:** Blood vessel decellularization, Biocompatibility, Arterial tissue engineering, Rabbit arteria carotis

## Abstract

**Background:**

The development of a suitable extracellular matrix (ECM) scaffold is the first step in vascular tissue engineering (VTE). Synthetic vascular grafts are available as an alternative to autologous vessels in large-diameter arteries (>8 mm) and medium-diameter arteries (6–8 mm). In small-diameter vessels (<6 mm), synthetic vascular grafts are of limited use due to poor patency rates. Compared with a vascular prosthesis, natural tissue ECM has valuable advantages. Despite considerable progress in recent years, identifying an optimal protocol to create a scaffold for use in small-diameter (<6 mm) fully natural tissue-engineered vascular grafts (TEVG), remains elusive. Although reports on different decellularization techniques have been numerous, combination of and comparison between these methods are scarce; therefore, we have compared five different decellularization protocols for making small-diameter (<6 mm) ECM scaffolds and evaluated their characteristics relative to those of fresh vascular controls.

**Results:**

The protocols differed in the choice of enzymatic digestion solvent, the use of non-ionic detergent, the durations of the individual steps, and UV crosslinking. Due to their small diameter and ready availability, rabbit arteria carotis were used as the source of the ECM scaffolds. The scaffolds were subcutaneously implanted in rats and the results were evaluated using various microscopy and immunostaining techniques.

**Conclusions:**

Our findings showed that a 2 h digestion time with 1× EDTA, replacing non-ionic detergent with double-distilled water for rinsing and the application of UV crosslinking gave rise to an ECM scaffold with the highest biocompatibility, lowest cytotoxicity and best mechanical properties for use in vivo or in situ pre-clinical research in VTE in comparison.

**Electronic supplementary material:**

The online version of this article (doi:10.1186/s12938-017-0344-9) contains supplementary material, which is available to authorized users.

## Background

Over 600,000 vascular grafts are implanted annually to replace damaged blood vessels, along with a considerable number of cardiac valves [1, 2]; therefore, there is increasing need to develop a tissue-engineered blood vessel substitute for use in cardiovascular tissue engineering [2, 3]. Despite rapid progress in recent years [2, 4], the critical factors to be overcome in vascular tissue engineering (VTE) technology are the same as those in other organ tissue engineering techniques; namely, selecting a suitable scaffold, seeding cells into it, and optimizing the microenvironment to ensure autonomous development of body-compatible tissue [2]. So far, the priorities in VTE have been identifying an ideal material for the scaffolds, finding the most suitable sources for seeding cells, and perfecting in vitro culturing conditions to create an optimal tissue-engineered vascular graft (TEVG).

The first TEVG was developed by Weinberg and Bell [5] in 1986 using collagen gels to construct a scaffold onto which endothelial cells, fibroblasts and smooth muscle cells were added. Since then, a series of artificial materials have been used to produce scaffolds for clinical trials [6–9]: Synthetic materials have achieved some success in replacing larger coronary vessels (6–10 mm in diameter) [10]; however, in vessels with diameters <6 mm, thrombotic events have rapidly led to the vessels being closed off [11, 12]. For smaller diameter arteries (typically, 3–4 mm) physicians have almost exclusively relied on autologous vein grafts (e.g. saphenous vein) [4, 13, 14]. Therefore, intense research is being conducted to create clinically acceptable, small diameter vascular prostheses as alternatives to autologous arterial or venal excisions, especially in cardiovascular bypass surgery [15].

In order to perform VTE in human patients, it is of utmost importance that physicians, scientists, regulators and engineers work in concert to ensure the safety and well-being of human subjects enrolled in clinical trials. The most impressive results to date have been achieved by using fully natural TEVGs [16]; however, research to find a suitable extracellular matrix (ECM) scaffold for these types of grafts continues [16]. Some research has reported using porcine, goat and bovine tissue for TEVGs with diameters of 4–6 mm [6, 17, 18]. However, studies on ECMs for use in blood vessels <2 mm in diameter are limited [15, 19–23], and reports on the effectiveness of different decellularization protocols are not abundant enough [20, 24–26]. For vessels in this size range, research has focused on the use of high molecular weight synthetic materials [27–30]. To achieve this, we selected rabbit arteria carotis, as opposed to synthetic materials, as the source of our scaffolds. The decellularization process is a compromise and balance between removal of cellular material and maintenance of the ECM. Further, considering the rationales for using different decellularization agents and their effects [17, 31], we conducted a series of tests to compare five different decellularization protocols and characterized the resulting scaffolds to identify an optimal method for creating an ECM scaffold with good mechanical properties and high biocompatibility for use in pre-clinical research.

## Methods

### Animals, harvesting of vascular samples and implantation of decellularized scaffolds

The animals were obtained from Xiamen University Animal Center. All the animals were anesthetized prior to extraction or implantation of vessels and scaffolds, and were humanely euthanized after the procedures were completed. The animal experiments were approved by the Animal Center of Xiamen University (Xiamen, China) and complied with the Instructive Notions with Respect to Caring for Laboratory Animals, 2006, published in by the Science and Technology Department of China.

Carotid arteries (20–40 mm in length with lumen diameters of 1.8–2.2 mm) were harvested from New Zealand white rabbits (male; 6 months old; weight, 2.0–2.5 kg). Warm ischemic time between tissue extraction to processing or storage at −20 °C was <1 h. Excess connective tissue was removed from the adventitia of the carotid arteries with a scalpel prior to decellularization.

Once decellularized, the scaffolds (length: 5 mm) were subcutaneously implanted into Sprague–Dawley rats (male; 4–6 weeks-old; weight 150 g). We used the suture line (6-0, PERMA-HAND Silk Suture; ETHICON INC., USA) which was used for locating the scaffolds as well as a maker to identify them when they were harvested. We implanted four samples in one group/rat at a spacing of 1.5 cm from each other. We harvested at the correct time point and maintained the samples of one group in the same background. For each set, we repeated this procedure thrice. The grafts were harvested on days 7, 14, 28 and 56 after implantation for histological examination and biocompatibility analysis. C57/B6 spleens were used as positive controls (Additional file 1: Figure S3) for in vivo studies, which are related to the immunofluorescent staining images shown in Fig. 4.

### Decellularization of vascular-scaffolds

The basic method in this research was the protocol for decellularization employed by Zhao et al. [17], and all improvements in the protocol have stemmed from it. The samples were divided into five groups (I–V), each corresponding to a different decellularization procedure. A sixth group was prepared as a negative control. The steps for the five experimental protocols are summarized in Table 1. The protocols differed in terms of the digestion solvent (pepsin dissolved in EDTA at two different concentrations), digestion time (1, 1.5 or 2 h), rinsing solution (double-distilled water or 1% Triton X100) and rinsing time (1 or 2 h). In addition, groups IV and V were subjected to ultraviolet irradiation for 30 min to promote crosslinking of the scaffold. The negative control was prepared by immersing of fresh vascular samples in double-distilled water (ddw) on an orbital shaker throughout course. After removal of the cellular components, the scaffolds were stored in PBS with 1% penicillin–streptomycin. The reaction temperature was maintained at 4 °C throughout all of the protocols (n = 5 for each group, repeated six times in all).

**Table 1:** Experimental details for the five different decellularization protocols

Protocol step	Group				
	I	II	III	IV	V
Soaking in ddw for 24 h	√	√	√	√	√
Frozen at −80 °C and thawed twice	√	√	√	√	√
Soaking: 75% ethanol Solvent:tissue ratio: 20:1 (v/w) Total duration: 72 h Solvent replacement at 1, 3, 6, 12, 24, 72 h	√	√	√	√	√
Soaking in ddw for 24 h	√	√	√	√	√
Digestion on an orbital shaker using a tissue:enzyme ratio (g/ml) of 1:15	0.125% pepsin	0.125% pepsin	0.125% pepsin	0.125% pepsin	0.125% pepsin
Solvent	1× EDTA	2× EDTA	1× EDTA	1× EDTA	2× EDTA
Duration (h)	1.5	1	2	2	1.5
PBS washing for 30 min; shaking	√	√	√	√	√
DNase and RNase for 6 h; shaking	√	√	√	√	√
Rinsing solution	ddw	ddw	Triton	ddw	Triton
Duration (h)	1	1	2	1	2
Ultraviolet irradiation for 30 min				√	√

DNase and RNase concentration was 70 U/ml, respectively

Each step was performed at 4 °C

n = 5 for each group, repeated six times in all

*ddw* double-distilled water, *Triton* 1% Triton X100

### Scanning electron microscopy (SEM)

The scaffolds were prepared for scanning electron microscopy (SEM) by fixing in 2.5% glutaraldehyde for 2 h followed by dehydration through a graded series of ethanol concentrations. The scaffolds were immersed in pure *tert*-butyl alcohol (TBA) overnight and air-dried for 24 h. The scaffolds were sputter-coated with gold prior to visualization using a JEOL JSM-6390LV SEM (Jeol; Japan).

### Histological analysis

The decellularized scaffolds from each group were harvested from the rats 7, 14, 28 and 56 days after implantation. The samples were washed in normal saline and fixed in 4% paraformaldehyde for 24 h before being dehydrated through a graded series of ethanol. The samples were then embedded in paraffin and sliced into 5 μm-thick sections. For histological identification of cellular material, the sections were stained with hematoxylin and eosin (H&E), 4,6-diamidino-2-phenylindole (DAPI), Masson’s trichrome (Maxim Inc.; China) or Picrosirius red (Microherb Inc.; China).

Immunofluorescent staining was performed using biotin conjugated anti-mouse CD3e and CD11b (1:250, 2 µg/ml, eBioscience; San Diego, CA, USA) primary antibodies, followed by streptavidin-HRP, goat-anti-mouse Alexa Fluor 488 (1:1000, 2 µg/ml, Invitrogen; Carlsbad, CA, US) secondary antibodies. DAPI was used as a nuclear counterstain. The stained samples were visualized using a Motic BA310 microscope and analyzed by Motic Image Advanced 3.2. software (Motic Asia; China). The thicknesses of the intima & media membranes were also measured.

### MTS cell proliferation assay

The MTS cell proliferation assay was performed using a CellTiter 96 AQueous One Solution Cell Proliferation Assay System (Promega; Madison, WI, USA). Briefly, the scaffolds were cultured in Dulbecco’s Modified Eagle Medium Nutrient Mixture F-12 (DMEM/F12) at 37 °C for 24 h at a density of 2.5 ml/cm^2^. The culture media (leach liquor) were collected and preserved. Human umbilical vein endothelial cells (HUVEC; ATCC; Rockville, MD, USA) were cultured to logarithmic phase, plated in 96-well plates (500 cells/well) and cultured for a further 24 h. The media were replaced with leach liquor from the scaffold cultures diluted with DMEM/F12 at the following ratios of 1:2, 1:4 and 1:8. The cells were cultured for a further 1, 3 and 5 days at 37 °C and their ODs were measured at 490 nm. A negative control was prepared using DMEM/F12 alone. The cytotoxicity of each protocol was evaluated by calculating the relative growth rate (RGR) [RGR = (mean OD for each group)/(mean OD of the negative control) × 100%] to determine the proliferation index. The evaluation standard was derived from the Biological evaluation of medical devices-Part 5: Test for in vitro cytotoxicity (GB/T 16886.5-2003/ISO 10993-5:1999, IDT).

### Mechanical testing of scaffolds

#### Burst-pressure testing of the scaffolds

Decellularized scaffolds (approximately 10 mm in length) were randomly selected from each group. Each scaffold was attached via tapered adapters directly into a pressurized tube. The burst pressures were measured by delivering normal saline from a 50 ml syringe via a control valve. The fluid pressure (mbar) was monitored using a manometer with a data acquisition system (RM6240BT; Chengdu Science Instrument Factory; China). The system was progressively and manually pressurized until fluid was observed erupting from the vessel. This was defined as vessel failure or the burst pressure.

#### Testing suture retention strength of the scaffolds

Suture strength was measured using a magnetic base three-dimensional micro thruster and data acquisition system (STW-2 and RM6240BT, respectively; Chengdu Science Instrument Factory) following the manufacture’s instructions. Briefly, vascular samples, approximately 20 mm in length, were chosen randomly; 5-0 polypropylene sutures were placed in each side of the scaffold (1 mm from the edge) and constant elongation (2 mm/s) was applied along the longitudinal axis of the scaffold until the sutures were pulled through the vessel edge; the maximum suture strength was then recorded. The procedure was repeated in each of the five experimental groups and the results were compared to the suture retention strength of the negative control.

### Statistical analysis

GraphPad Prism v. 5.0 software (GraphPad Inc.; San Diego, CA, USA) was used for all the statistical analyses. Data were expressed as mean ± SD. One-way analysis of variance (ANOVA) was performed to test for statistical differences. The burst-pressure test, suture retention test and intima & media thickness measurements were repeated three times in each group. The figures show the results from the final trials. A *p* value <0.05 was considered statistically significant.

## Results

### Overall appearances of the scaffolds

**Fig. 1:**
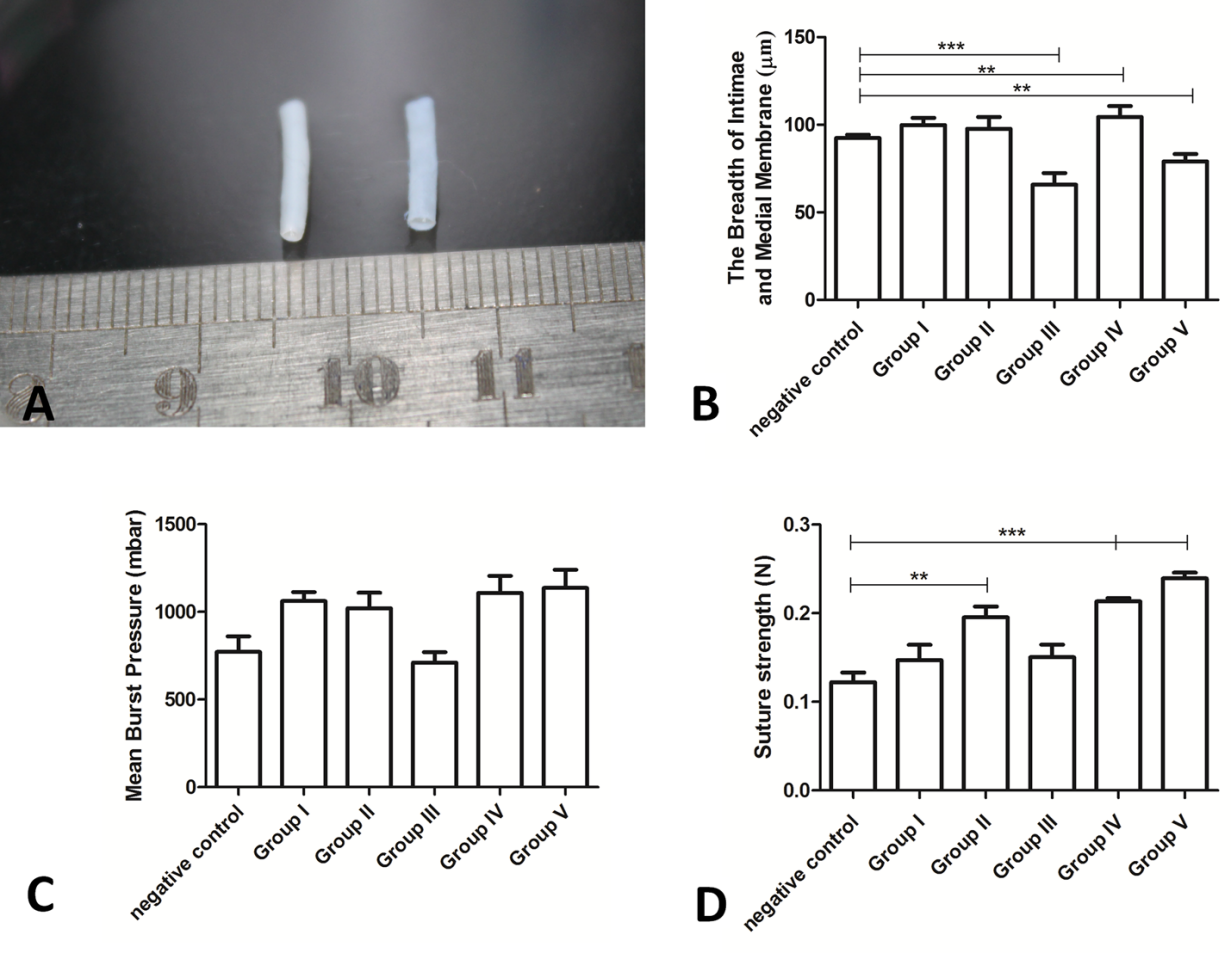
Characterization of decellularized blood vessel scaffolds. **A** A gross view of fresh (*left*) and decellularized (*right*) rabbit carotid artery (length, 10 mm; internal diameter, 1.8 mm). Data comparisons between the following characteristics of decellularized scaffolds prepared by five different protocols (I–V) and fresh vascular controls; **B** breadths of the intima and media membranes (n = 6); **C** mean burst pressures (n = 6); **D** suture retention strengths (n = 6). **p < 0.01; ***p < 0.001

Images of decellularized blood vessel scaffolds are shown in Fig. 1A. The decellularized scaffold (right) was chosen randomly from among the experimental groups, whereas the negative control vessel shown on the left was treated by ddw only. We also measured the breadth of intimae and medial membranes. This measurement was done on images of all three histological stains (n = 3) by Motic Image Advanced v3.2 (Motic Asia, Beijing, China) (Fig. 1B). Statistical analyses for breadth between the groups revealed significant differences in the decellularized scaffolds in groups IV and V compared with those in the control group (*p* < 0.01), especially for group III versus the control group (*p* < 0.001). However, no significant differences were observed in the thicknesses of the intima and media between groups I and II *versus* the control group.

**Fig. 2:**
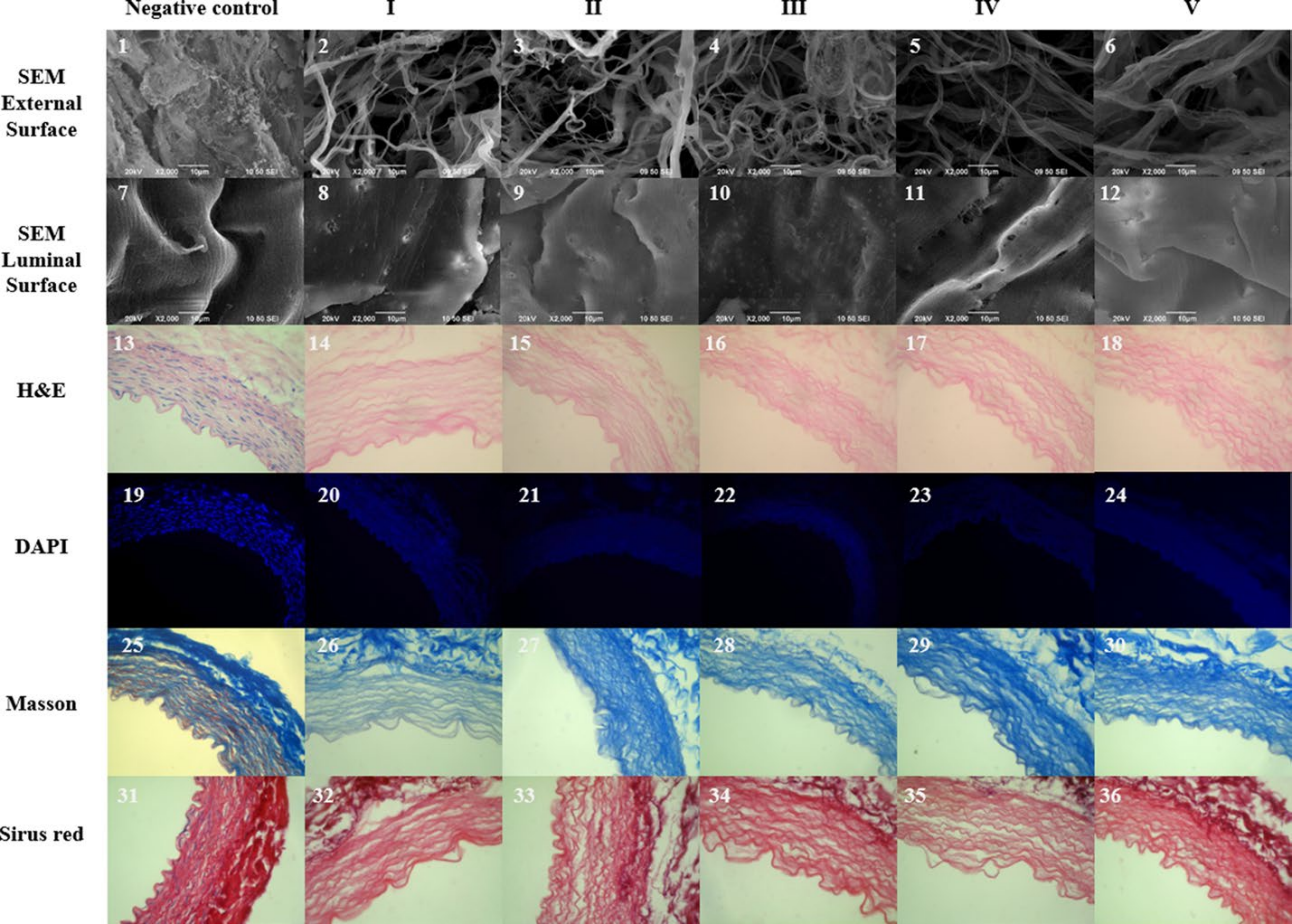
Visual characterization of decellularized blood vessel scaffolds. Various microscopic and histological staining techniques were employed to compare the following characteristics between five experimental decellularized scaffolds and fresh vascular controls. (*1*–*6*) Scanning electron micrographs (SEM) showing the external surface appearances (magnification, ×2000) and (*7*–*12*) the luminal surface appearances of the scaffolds (magnification, ×2000); (*13*–*18*) hematoxylin and eosin (H&E) staining (magnification, ×400) and (*19*–*24*) DAPI staining (magnification, ×200) showing cross-sections of the scaffolds and the presence of nuclear residue; (*25*–*30*) Masson’s trichrome staining results (magnification, ×400) and (*31*–*36*) Picrosirius red staining showing the structures of the pores and collagen fibers in the matrices of the scaffolds (magnification, ×400)

SEM examination of the ultrastructure of the scaffolds showed that the general appearances of the matrices in the five experimental groups were similar: the microporous structures on the outside of the matrices were sufficiently open for migration and ingrowth of seeded cells, and all of the experimental scaffolds were more porous than the control vessels (Fig. 2; [1–6]). There was little evidence of cellular residue on the surfaces of the matrices in groups IV and V, however, a small amount of cell residue was visible on the surfaces of the scaffolds in groups I, II and III. The appearance could be observed in luminal surface images (Fig. 2; [7–12]). In comparison, the luminal surfaces in group III and IV were more porous than those for other groups. Also, the appearance in group III was not so clear as for group IV.

### Histological analysis of the decellularized scaffolds

The cross-sections of the scaffolds in each group were examined by H&E staining (Fig. 2; [13–18]) and DAPI staining (Fig. 2; [19–24]). Although nuclear residue was still evident in the control group (Fig. 2; [13]), almost no nuclear material was visible in the five decellularized scaffolds. This was confirmed though DNA content analysis (Additional file 1: Figure S1).

Masson’s trichrome staining and picrosirius red staining revealed that the pores in each of the experimental scaffolds were intersected with small collagen fibers resulting in small pore sizes, with tight packing of the large collagen-fiber network and elastin layers, producing a tight, dense matrix (Fig. 2; [25–36]). Blue showed collagen and elastin in the scaffolds upon Masson’s trichrome staining. Dark-red showed collagenous fibers in the scaffolds with picrosirius red staining.

### Mechanical properties

#### Burst pressure testing of decellularized scaffolds

In general, the burst pressures of the decellularized scaffolds in the experimental groups were greater than those in the control group; however none of the differences were significant (Fig. 1C). The greatest differences were observed between groups IV and V versus the control group, both of these groups had been subjected to UV crosslinking. Conversely, a small, non-significant decrease was observed in the burst pressure of the scaffolds in group III compared to the control vessels.

#### Suture retention strength of the scaffolds

The retention strength of the sutures prior to implantation was not statistically different to that recorded in native rabbit carotid arteries, suggesting that the vascular graft scaffold had sufficient suture retention strength to withstand anastomotic forces. Suture retention analysis between the groups revealed significant differences in the retention strengths in the decellularized scaffolds in groups II, IV and V compared to those in the control group (Fig. 1D; *p* < 0.01), especially in groups IV and V (*p* < 0.001).

### Biocompatibility of decellularized scaffolds in vitro and in vivo

#### Cytotoxicity of the decellularized scaffolds in vitro

The cytotoxicities of the decellularized scaffolds were determined by MTS assay, as previously described [32]. The RGRs of HUVECs grown in the presence of leach liquor from the scaffolds at different concentrations after 1, 3 and 5 days of culture were evaluated (Additional file 1: Figure S2). On day 1, no apparent differences were observed in the RGRs between the experimental groups and the control group; on days 3 and 5, the RGRs in the experiment groups were lower than that in the control group, However the differences remained non-significant (Additional file 1: Figure S2). Considering the rate and density of cell growth, we determined that the RGRs on day 3 of co-culture were most suitable for detailed analyses, and these results are summarized in Table 2. Group I, II, IV were level 1 and their RGR was 78.05, 88.22 and 77.24%, respectively. Group III and V were level 2, and their RGR was 72.40 and 60.29%, respectively. Low cytotoxicity (level 1) was defined as RGR ≥75%, and medium cytotoxicity was defined as 50% ≤ RGR ≥ 74% (level 2). The evaluation standard was derived from the *Biological evaluation of medical devices*—*part 5: test for* in vitro *cytotoxicity* (GB/T 16886.5-2003/ISO 10993-5:1999, IDT).

**Table 2:** Evaluation of scaffold cytotoxicity in vitro for each protocol

Group	OD value (mean ± SD)	RGR (%)	Level
Negative control	1.239 ± 0.04	100	
Group I	0.967 ± 0.02	78.05	1
Group II	1.093 ± 0.05	88.22	1
Group III	0.897 ± 0.03	72.40	2
Group IV	0.957 ± 0.04	77.24	1
Group V	0.747 ± 0.01	60.29	2

Cytotoxicity evaluation: RGR: relative growth ratio; level 1: RGR = 75–99%, low cytotoxicity; level 2: RGR = 50–74%, medium cytotoxicity

The evaluation standard was derived from the *Biological evaluation of medical devices*—*part 5: test for* in vitro *cytotoxicity* (GB/T 16886.5-2003/ISO 10993-5:1999, IDT)

#### Host cell infiltration and integration of the decellularized scaffolds in vivo

**Fig. 3:**
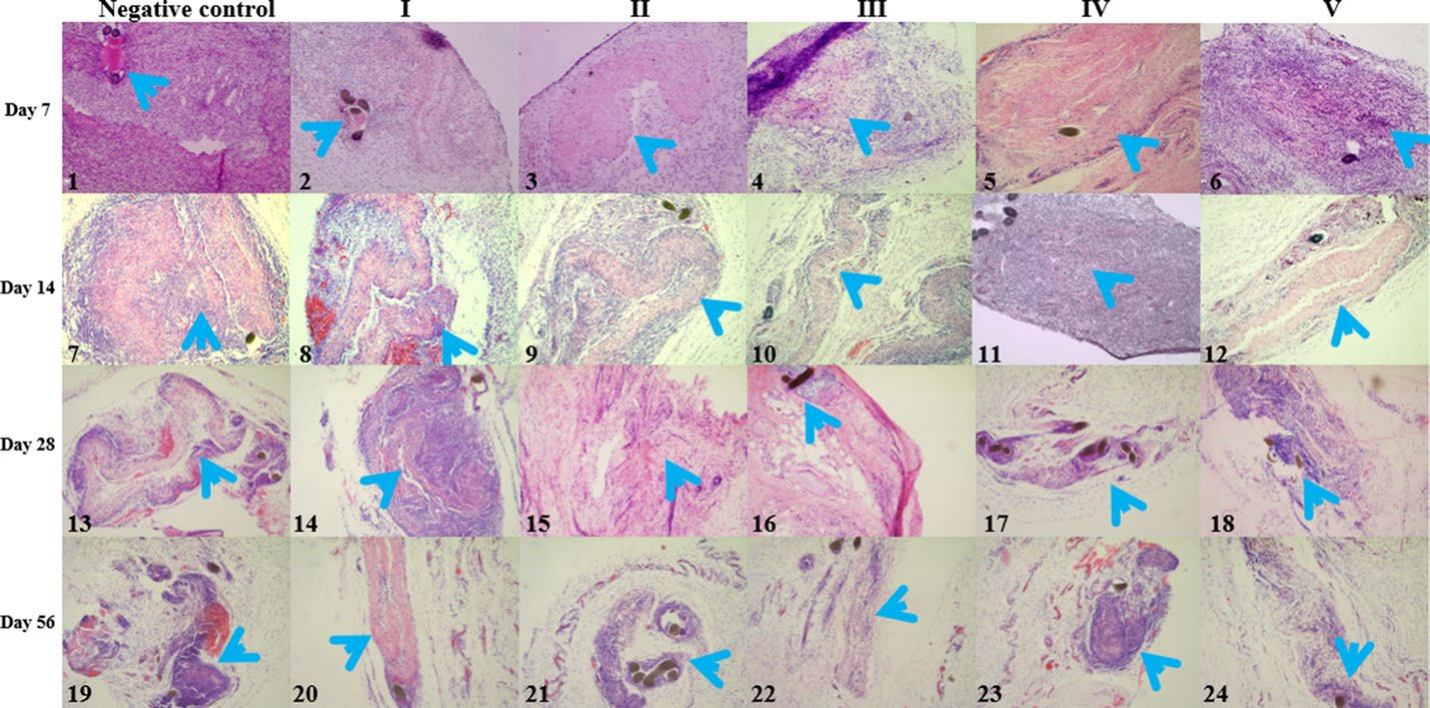
Biocompatibility of decellularized blood vessel scaffolds in vivo. H&E staining showing host cell infiltration in decellularized scaffolds from rats at various time points following subcutaneous implantation compared to fresh vascular controls: day 7 (*1*–*6*); day 14 (*7*–*12*); day 28 (*13*–*18*); and day 56 (*19*–*24*). The images show progressive degradation and a decrease in inflammatory infiltration in the experimental scaffolds over time. In contrast, the extent of inflammatory infiltration in the control sample remains high throughout, with a clear boundary separating the scaffolds from the receptor tissue. At day 56, there is almost no evidence of the scaffold in the experimental groups. The *blue arrow* indicates the scaffold position; the suture points are identified by a *dark grey point* (magnification, ×100)

The grafts were harvested on days 7, 14, 28 and 56 after subcutaneous implantation of the scaffolds in Sprague–Dawley rats. H&E examination of the control sections showed that a large number of cells had embraced the graft 7 days after implantation with a clear boundary separating the scaffold from the receptor tissue (Fig. 3; [1]) until day 56 (Fig. 3; [7, 13, 19]). In the experimental groups, infiltrations of host cells into the grafts were observed (Fig. 3; [2–6]). Especially for groups II and V, the host cell infiltration was less. Furthermore, these levels progressively increased in the experimental groups and by day 14, the boundaries between the experimental decellularized scaffolds and receptor tissues in the experimental groups had become vague (Fig. 3; [8–12]). In contrast, the infiltration boundary in the control group remained obvious. By day 28, the scaffolds in some of the groups had almost completely amalgamated into the surrounding tissue and could no longer be recognized (Fig. 3; [14–18]), but a clear boundary could be identified in the control group. The same identification could be confirmed on day 56 (Fig. 3; [19–24]). However, the images were varied (see below).

**Fig. 4:**
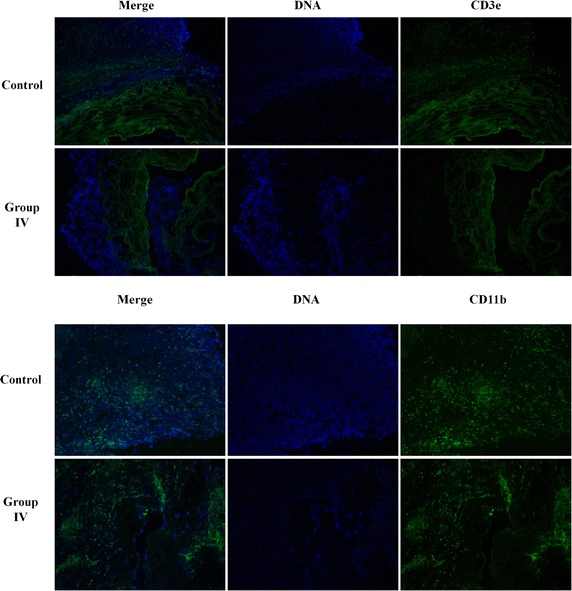
Immunoreaction in decellularized blood vessel scaffolds in vivo. The images compare the immunoreactivity in the scaffolds prepared via protocol IV (considered the optimal protocol of the five experimental trials) 7 days after subcutaneous implantation in rats against that in fresh vascular controls. The *green* fluorescence shows the distributions of CD3e and CD11b (markers of T cells and macrophage cells, respectively) within the samples (magnification, ×100)

Upon comprehensive comparison of all the data, group IV had shown the best performance, especially for the best breadth of intimae and medial membrane and ultrastructure according to SEM; therefore, this group was selected to assess the immunoreactivity of the decellularized scaffolds. We chose CD3e as a marker for T cells, whereas CD11b denoted macrophages. The results of the immunofluorescent assay (Fig. 4) showed a marked decrease in the intensity of CD3e in these scaffolds compared with that in the control group. Furthermore, the T cells only embraced the vessels, few were inside; there was also different expression in the intensity of CD11b between the two groups. The corresponding micrographs for the staining controls (marker) are given in Additional file 1: Figure S3.

## Discussion

Most research into TEVG has focused on the use of macromolecular synthetic materials, such as PGA [20, 21], polycaprolactone [27], polypropylene carbonate (PPC) [33], and electrospun tecophilic/gelatin nanofibers [34]. However, these yielded unsatisfactory long-term patency due to thrombotic failure in small-diameter vessels (<6 mm). The decellularized natural matrices took advantage of structure and mechanical performance, while avoiding adverse immunological reactions due to their origin [4]. As a consequence, research has increasingly been directed toward the suitability of fully natural vascular materials and ECM scaffolds prepared from blood vessels extracted from animals or human subjects [7, 9, 18, 35–39]. By combining these types of scaffolds with seeding cells obtained from the patient (autologous stem cells or endothelial cells), the TEVG forms an xenograft that has less thrombogenicity than synthetic materials and the characteristics required to promote cell attachment, migration and proliferation. Several groups have successfully developed xenogeneic bioprosthetic valves consisting of chemically crosslinked, intact porcine aortic valves, or valves created from crosslinked bovine pericardial tissue [40–42]; however, none of these methods have achieved vascular scaffolds with diameters ≤2 mm. In this study, we selected rabbit arteria carotis as the source of ECM scaffolds as these vessels have diameters of ~ 2 mm. Compared with using vessels from ovines [18, 43] and canines [24, 37], rabbits are easier to get and cost less; moreover, the vessel diameter of rabbits is nearly 2 mm, compared with around 4–6 mm from ovines and canines. Thus, rabbit is a suitable animal model for evaluation research of TEVG.

Initially, we prepared the scaffolds according to the protocol of Zhao et al. [17].; however, these achieved limited success, possibly due to a failure in fully removing cellular contamination, or due to factors associated with the surface active agent, rinse step, temperatures or durations, or because the rabbit arteria carotis was smaller than that from sheep. Furthermore, we considered the different effects of physical, chemical and biological agents and the convenience of operation in the laboratory setting [31]. With regard to physical agents, the temperature was the initial choice, which operated as freezing and thawing for several cycles. With respect to chemical agents, non-ionic detergents such as Triton X-100 [44] are more effective than ionic detergents such as deoxycholate [45, 46]. Meanwhile, biological agents (as pepsin, DNase and RNase) are commonly used for decellularization agent but must be followed by rinsing. Currently, a combination of several agents is popular in the decellularization process to avoid single side effects for only one agent [4]. Finally, we compared five different decellularization protocols using combinations of the agents mentioned above to identify the one that produced the cleanest ECM scaffold, with good mechanical properties, biocompatibility and minimal cytotoxicity, which are the combined potential beneficial effects of biological scaffolds. Each protocol differed in the digestion conditions for cell removal, rinsing conditions (non-ionic detergent vs. ddw) and whether the sample was subjected to UV irradiation to promote crosslinking, meanwhile avoiding potential cytotoxicity [47]. Even though we chose the agents, it remained difficult to ensure the concentration and time for each agent. As reported in our pilot experiment [17], we decided that the common sections were: (1) freezing at −80 °C and thawing twice; (2) soaking in 75% ethanol as mentioned in Table 1; (3) PBS washing for 30 min with shaking between pepsin and nucleases, (4) operation at 4 °C all the time. We set up different sections for each group: group I, 0.125% pepsin in commercial EDTA solvating agent (defined as 1× EDTA)/1.5 h shaking; group II, 2× EDTA/1 h shaking; group III 1× EDTA/2 h shaking and rinsing only in Triton X-100, not ddw; group IV 1× EDTA/2 h shaking and rinsing in ddw, addition of ultraviolet irradiation for 30 min; group V, 2× EDTA/1.5 h shaking and rinsing as group III, addition of ultraviolet irradiation for 30 min.

A negative control was prepared by immersing a fresh vascular sample in ddw throughout the protocol. DNase and RNase soaking for 6 h was sufficient for nucleic acid removal. Therefore, there was no different among experimental groups in terms of full infiltration, as reported previously [25, 38, 48].

Examination of the resulting scaffolds by SEM and histological microscopy showed there was little difference between the five protocols in relation to cellular residue. The ECMs prepared through protocols III and V showed the least smooth outer surfaces and rupture or tenuity of collagen could be observed according to Fig. 1B. Even both these protocols involved Triton X-100 in the rinsing step, we concluded that Triton X-100 could be a break factor for collagen structure and its cytotoxicity. This was confirmed by examining the intima and media through Masson’s trichrome and Picrosirius red staining. Furthermore, protocols III and V resulted in the highest levels of cytotoxicity (level 2), indicating that these scaffolds were unsuitable for clinical use. In contrast, the cytotoxicity of the remaining scaffolds (groups I, II and IV) was low (level 1) relative to the cytotoxicity in the control group. We speculated that the increased cytotoxicity in groups III and V was also a consequence of the Triton X-100 detergent. We could discard these two protocols for consideration. Preservation of the intima and media is important for the maintenance of cell function in decellularized scaffolds [20, 24, 26, 49]. Of the other three protocols, protocol IV yielded the tightest and least broken ECM structure with the thickest intima and media according to the SEM and breadth in Fig. 1B. The suture retention strengths of ECM scaffolds determine whether the grafts can withstand the forces exerted by anastomosis. The mechanical properties of the scaffolds were evaluated via burst pressure and suture retention strength tests. Testing of the burst pressure was in agreement with the results of Zhao et al. [17]. Although there were no statistically significant differences in the burst pressures between the experimental groups (Fig. 1C), there were significant differences in suture retention strength between groups II, IV and V and that of the control group. The differences were greatest with protocols IV and V (p < 0.001); whereas, protocol III showed the worst performance in both tests.

In order to establish whether decellularized scaffolds prepared from rabbit carotid arteries had sufficiently low immunogenicity for xeno-implantation, and thereby the ability to enhance tissue ingrowth in vivo, subcutaneous implantation of the decellularized scaffolds was performed in Sprague–Dawley rats and the results were examined through immunohistochemical staining. We observed a progressive reduction in inflammatory reactions between the experimental groups and the control group. The biodegradability of vascular scaffolds is a critical factor in their clinical suitability [49–51]. We found that after 56 days, the ECM scaffolds in groups II, IV and V had nearly disappeared and could only be recognized by their suture points; in contrast, the infiltration of inflammatory cells in group I and the control group was clearly evident. Degradation of the scaffolds was fastest in groups IV and V, both of which had undergone UV crosslinking reactions. We had discarded group III and V, so we finally obtained the protocol of group IV by comparison.

In combination, the findings suggested that the use of Triton X-100 in the rinsing step (III and V) and increasing the concentration of EDTA in the digestion step (II and V) both led to an increase in the pore size of the matrix; however, this effect could be reversed by crosslinking through UV irradiation (IV and V). This was consistent with a previous study carried out using porcine carotid arteries [52]. Overall, protocol IV appeared to be the most effective method for creating an ECM substitute for use in small-diameter cardiovascular vessels <2 mm. To confirm the biocompatibility of the scaffold prepared through this protocol, we employed CD3e and CD11b as markers for T cells and macrophage cells, respectively, by immunofluorescence (Fig. 4). In relation to CD3e (T cells), the results showed a clear reduction in the immunoreactivity of this scaffold compared to that in the control group, demonstrating that protocol IV possessed superior biocompatibility. Also, we observed that the T cells only embraced the scaffolds, with few inside, which may suggest that the immunological reaction was still activated. While there was also a reduction in the level of CD11b (macrophages), however the difference was small, and it showed that the marked cells in the control group were distributed compared with those in group IV. However, in group IV, the cells embraced the boundary of the scaffold, possibly due to the critical roles of macrophages in neovascularization and adipogenesis [49, 50, 53]. Of note, was the observation that the macrophages in group IV were localized around the scaffolds, indicating greater immigration; whereas no such localization was observed in the control group. Actually, it would be better to obtain all of the groups immunostaining images. It’s a limitation of this study.

Apart from what has been discussed above, other factors can influence the decellularization process [54], such as physical factors (rocking/whirling/sonicating) or device procedural factors. We developed a simple combination for decellularization, but three-dimensional printing technology is growing fast [55]. One of the limitations of our study was that we focused only on the preparation and characterization of small-diameter decellularized scaffolds as a common approach for pre-clinical research. More research must be done on the interaction between factors during decellularization.

## Conclusion

The ultimate goal of our study was to develop a small-diameter TEVG using a decellularized ECM scaffold sourced from rabbit carotid arteries which could be used as a model for pre-clinical research. Five protocols were tested and compared, each differing in the enzymatic digestion and rinsing conditions and the duration of the individual steps: the non-ionic detergent, Triton X-100 increased their cytotoxicity; however, suture strength could be rescued through UV crosslinking; and the rate of scaffold degradation could be increased by altering the concentration of EDTA used in enzymatic digestion.

Overall, in the five groups of this research, protocol IV produced a decellularized ECM scaffold with the highest biocompatibility, lowest cytotoxicity and the best mechanical properties. Hence, we provided a suitable protocol to make a small-diameter vessel scaffold model for preclinical research.

## Additional file

**Additional file 1.** Additional figures.
