# Supplementary material for: Preparation and characterization of small-diameter decellularized scaffolds for vascular tissue engineering in an animal model
Source: Biomed Eng Online. 2017 May 11;16:55. doi: 10.1186/s12938-017-0344-9 (PMC5425976; doi:10.1186/s12938-017-0344-9)
Supplement: Supplementary file 1 — Additional file 1. Additional figures. [file 12938_2017_344_MOESM1_ESM.doc]

Assessment and quantification of DNA

Total DNA was isolated from 25 mg of tissue (dry weight) using a PureLink® Genomic DNA Mini kit (Invitrogen, K182001). Then, DNA was quantiﬁed by measuring nucleic-acid concentration with a Quant-iT™ PicoGreen® dsDNA Reagent kit (Invitrogen, P7589) according to manufacturer instructions and normalized to the initial dry weight of samples.


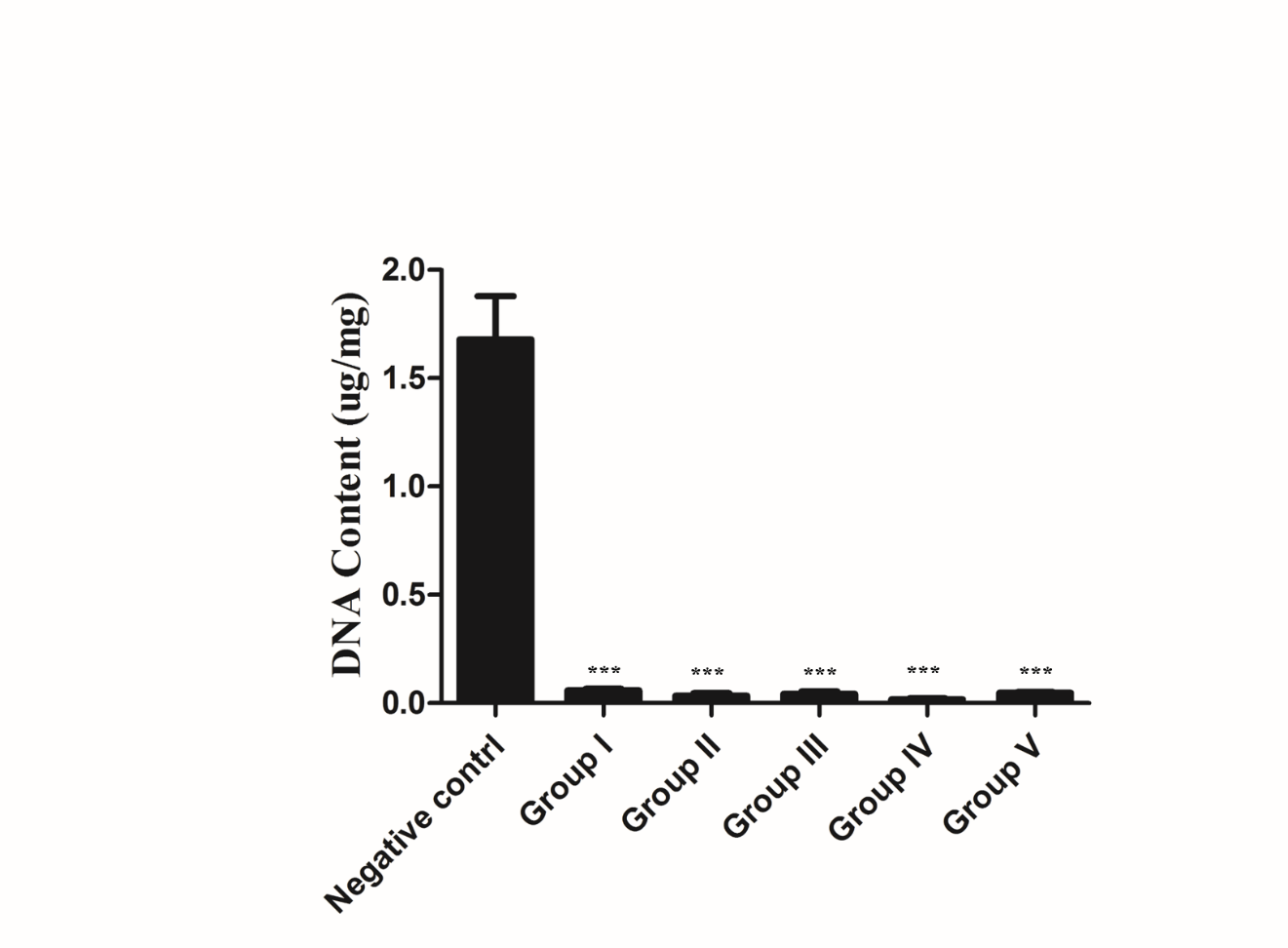


**Supplementary Fig. 1.** Residual DNA content examination. Data comparisons between decellularized scaffolds prepared by five different protocols (I-V) and fresh vascular control (negative control), n=3/group. ****p*<0.001.


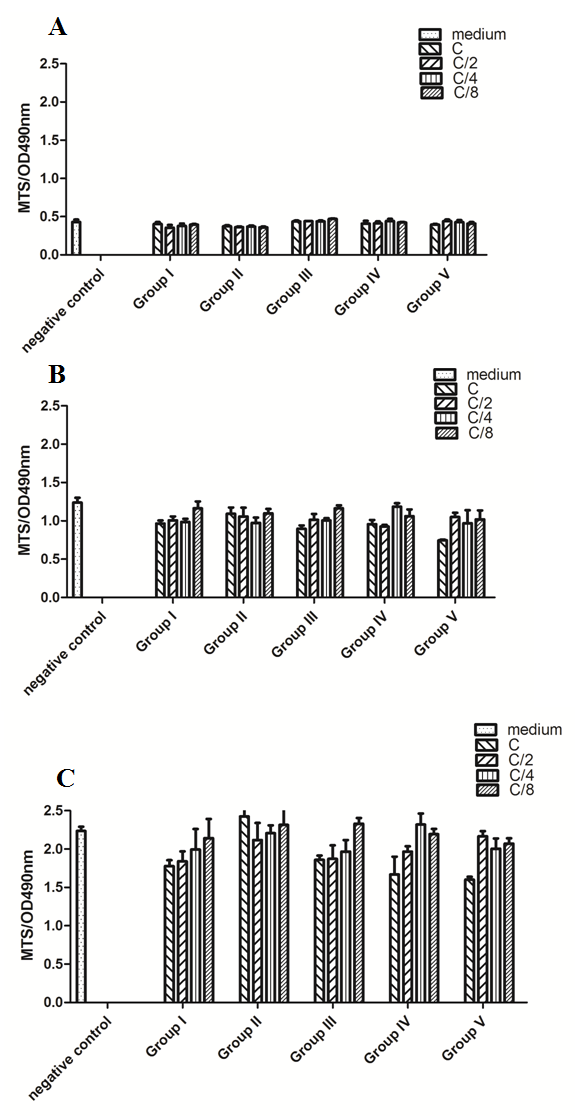


**Supplementary Fig. 2.** MTS assay showing relative growth rates(RGR). Proliferation of human umbilical vein endothelial cells (HUVEC) co-cultured with leach liquor from the different scaffolds (length, 10 mm; internal diameter, 1.8 mm) *in vitro* for one day(A), three days(B), and five days(C).


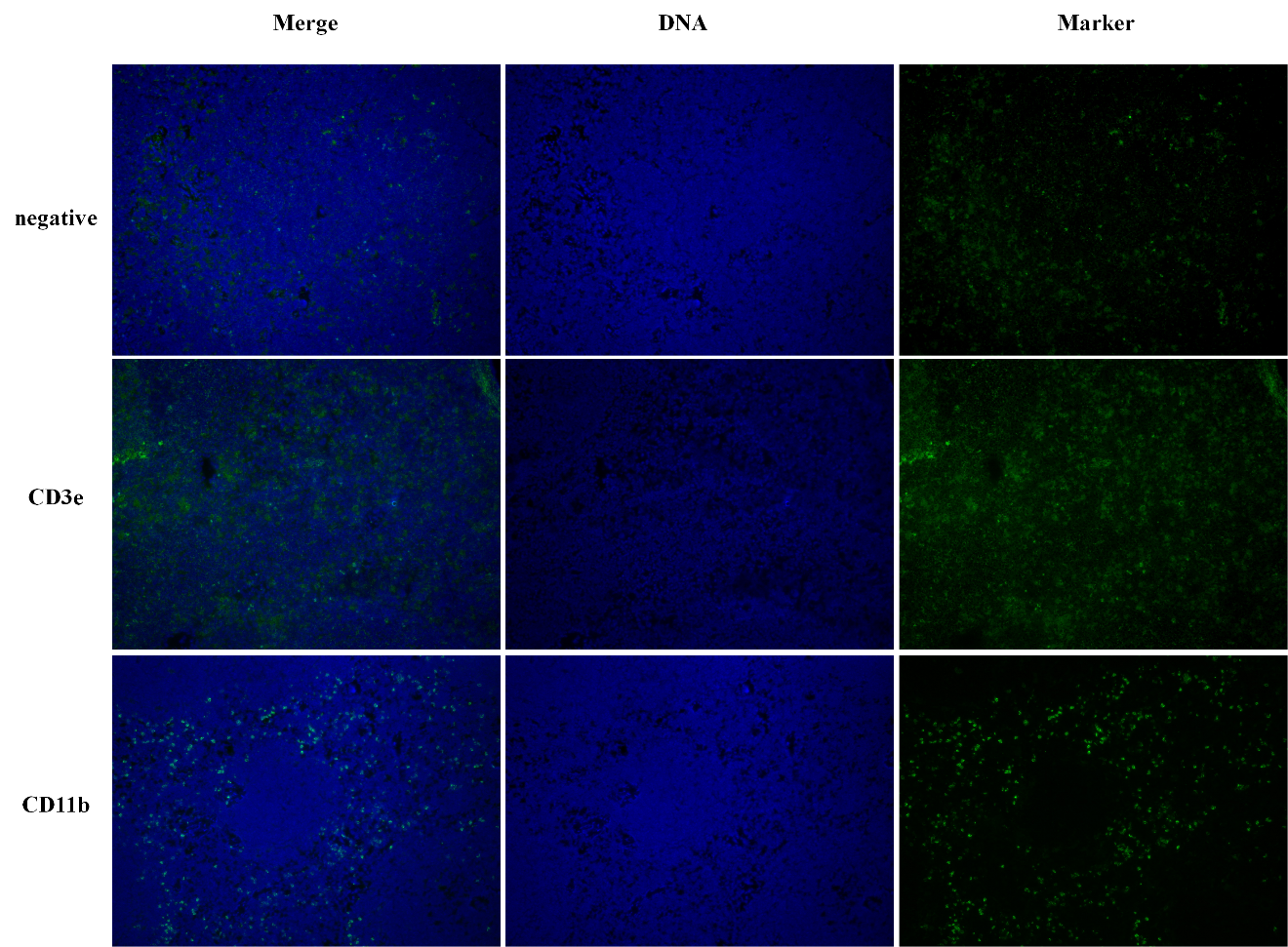


**Supplementary Fig. 3.** Negative and positive immunoreactivity controls on Day 7 after subcutaneous transplantation of the scaffolds in vivo. Tissu source: C57/B6 spleen. (magnification, 100x).


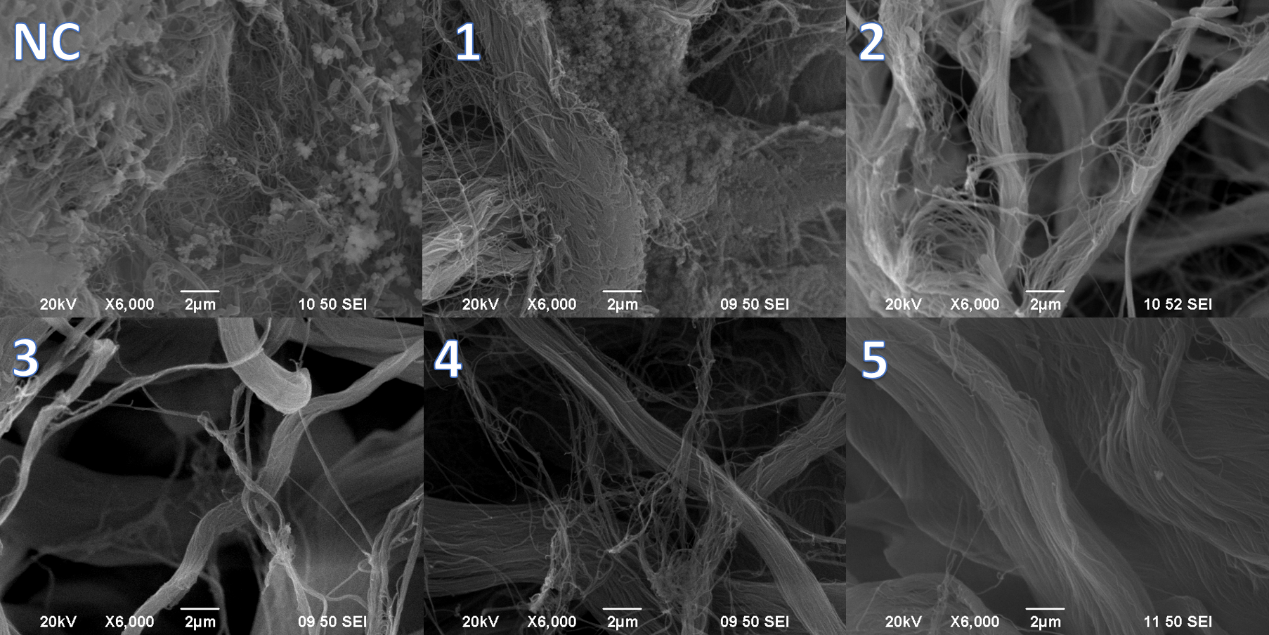


**Supplementary Fig. 4.** Scanning electron micrographs (SEM) showing the external surface appearances (magnification, 6000x) NC=negative control, the numbers stand for the related experimental group.
